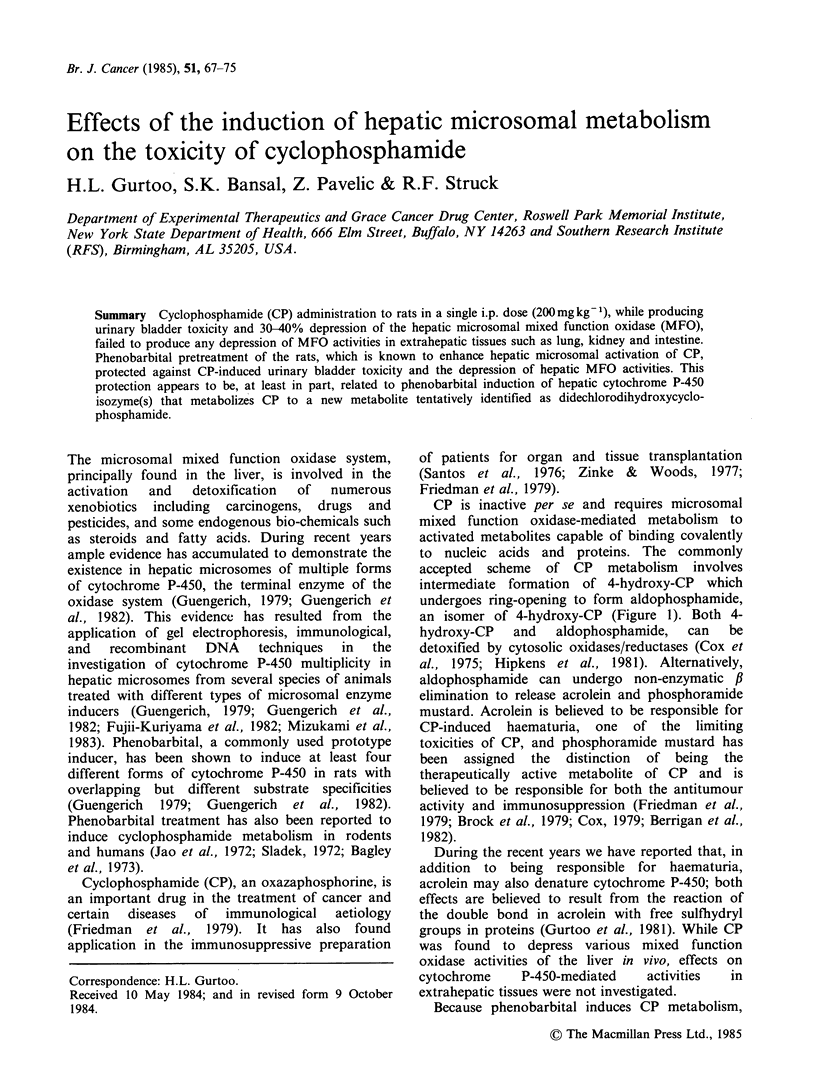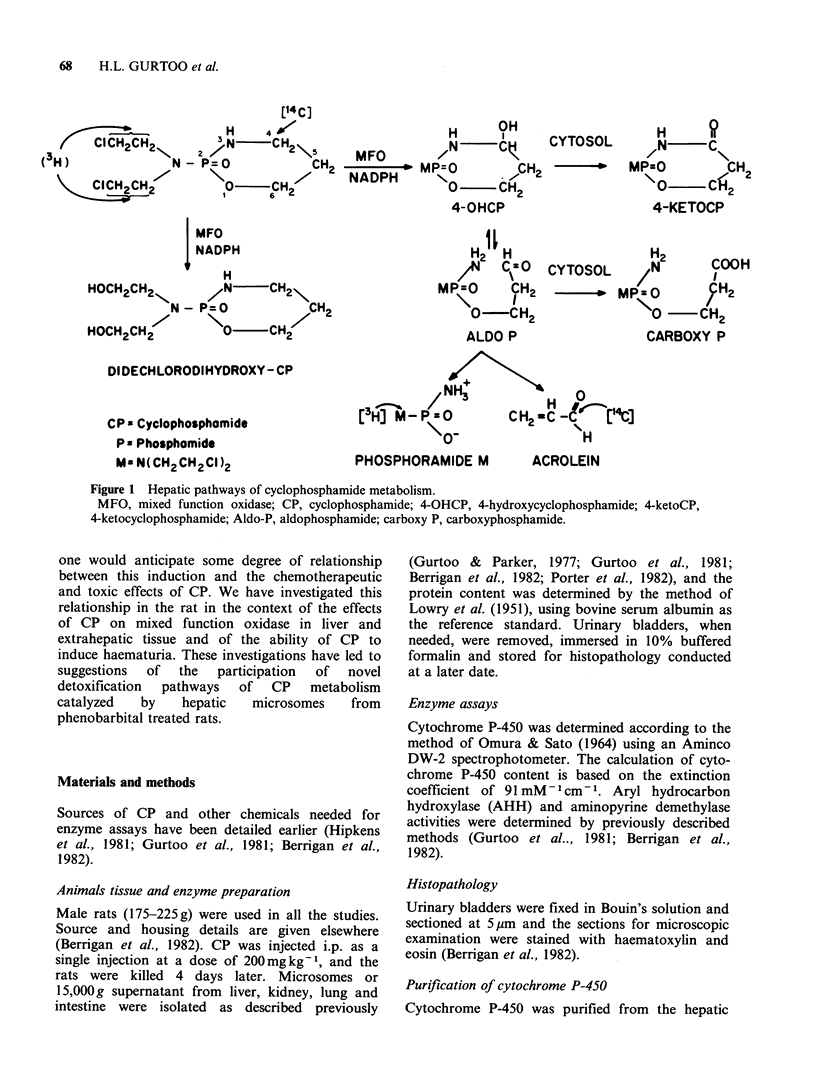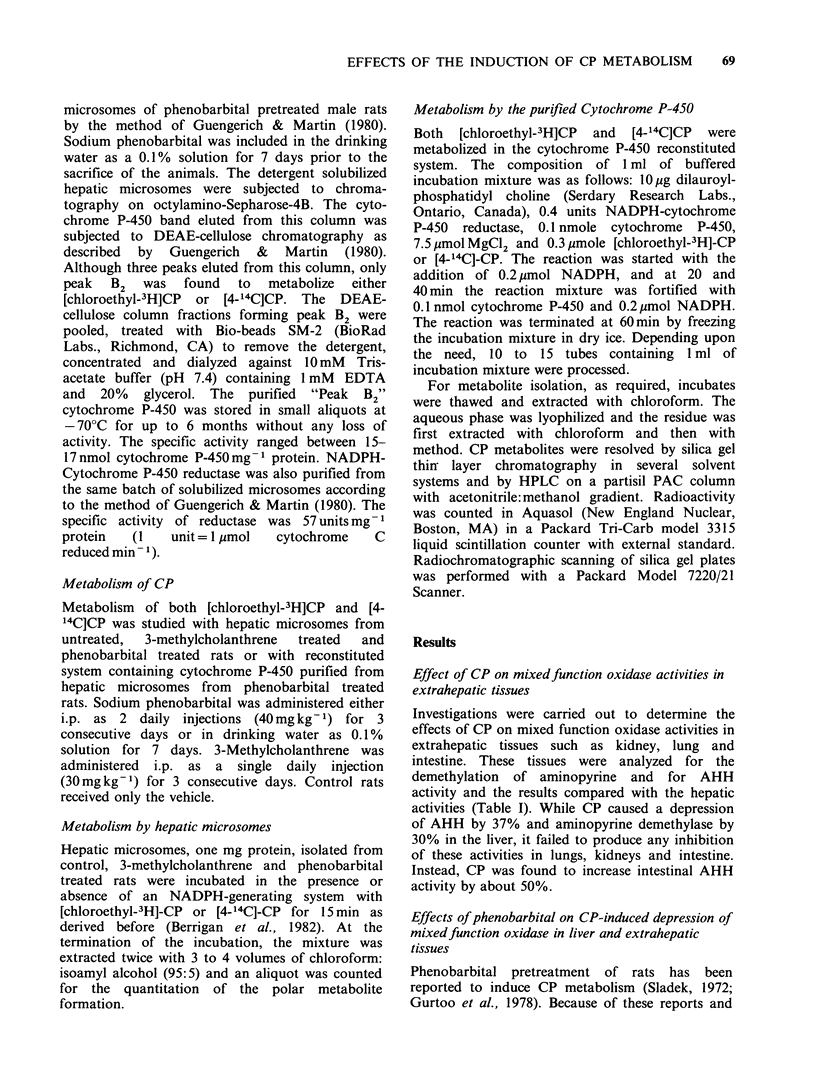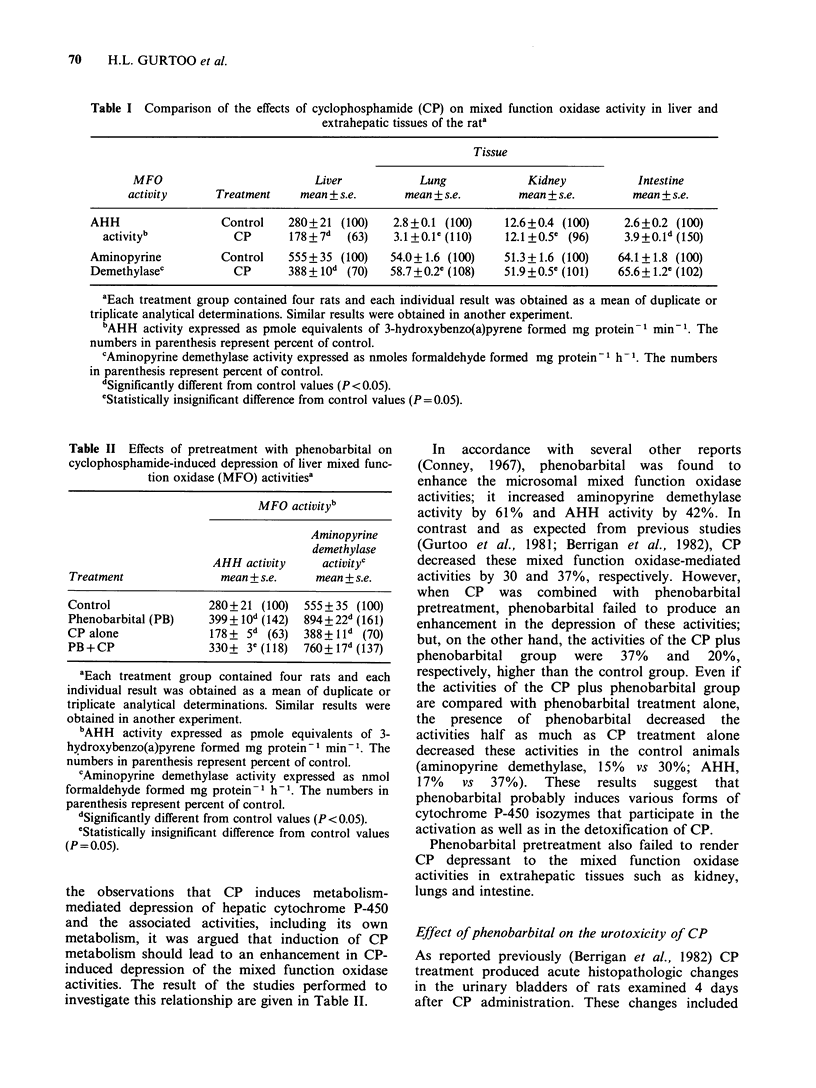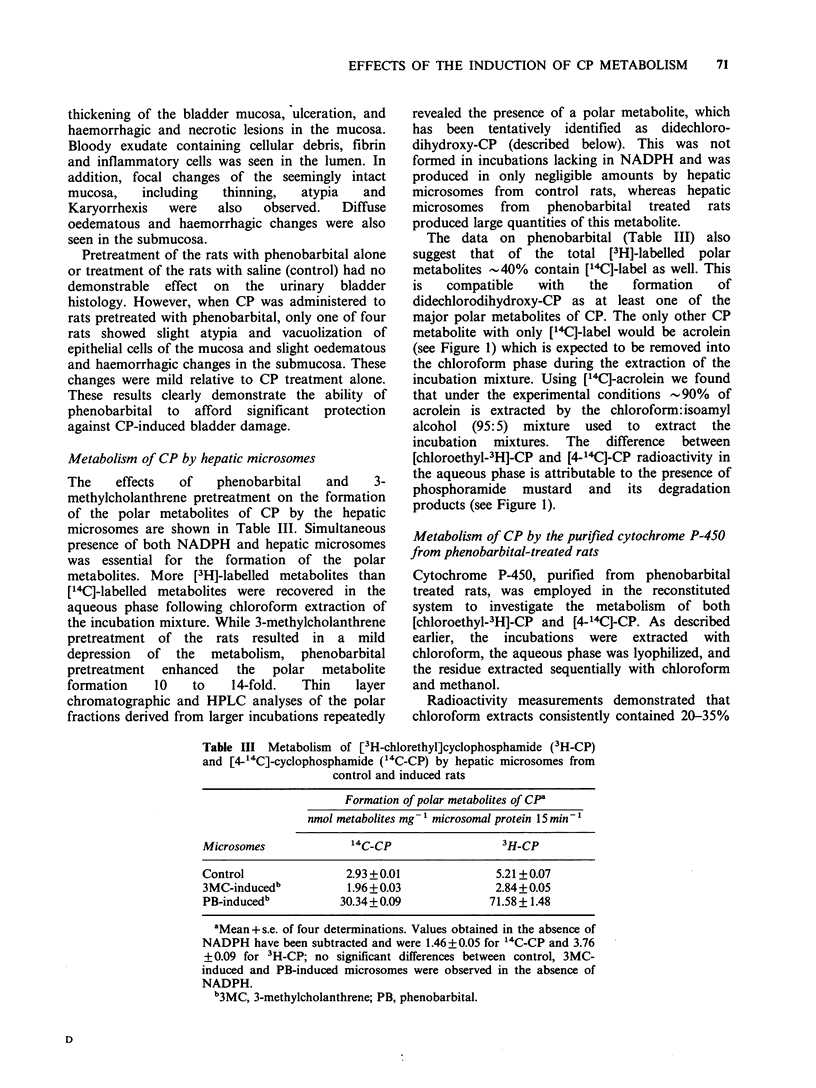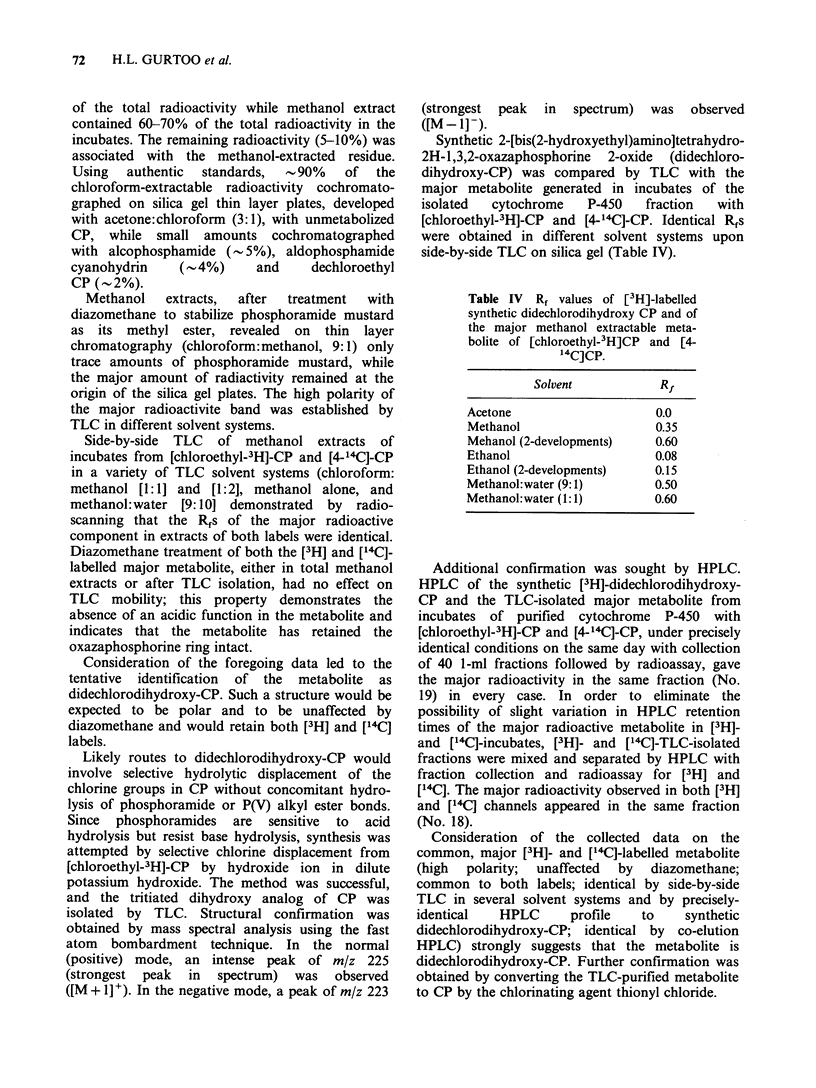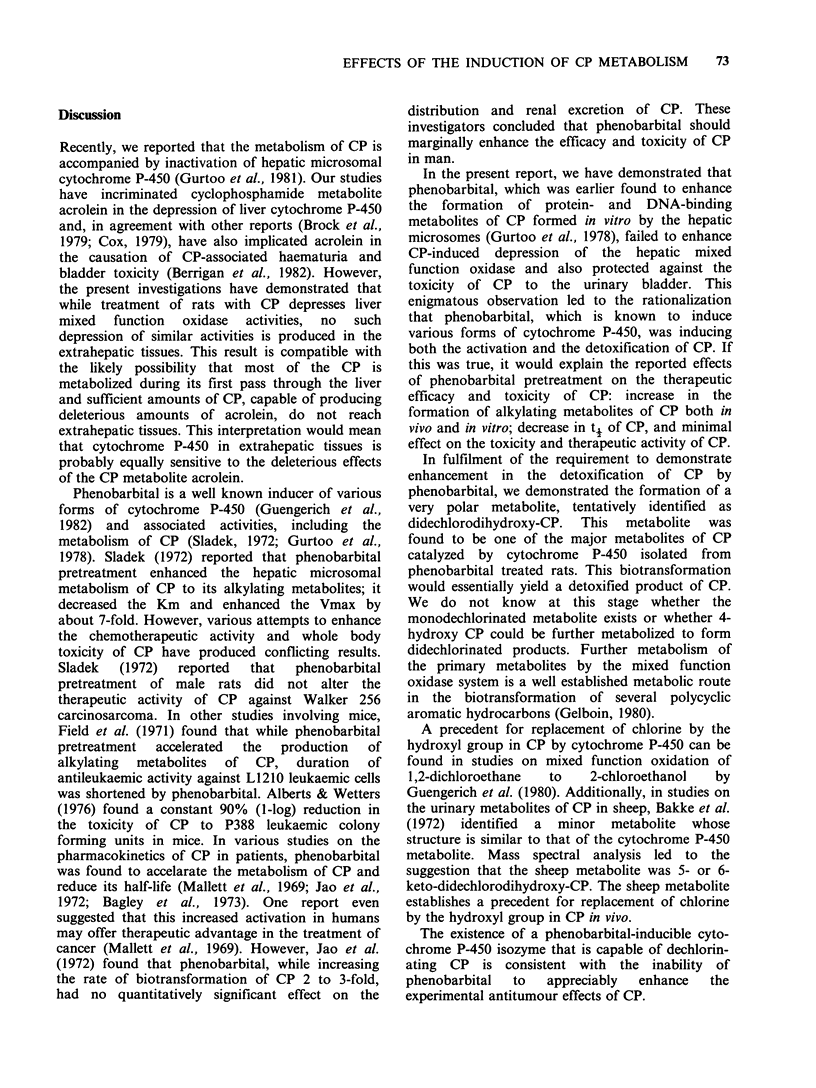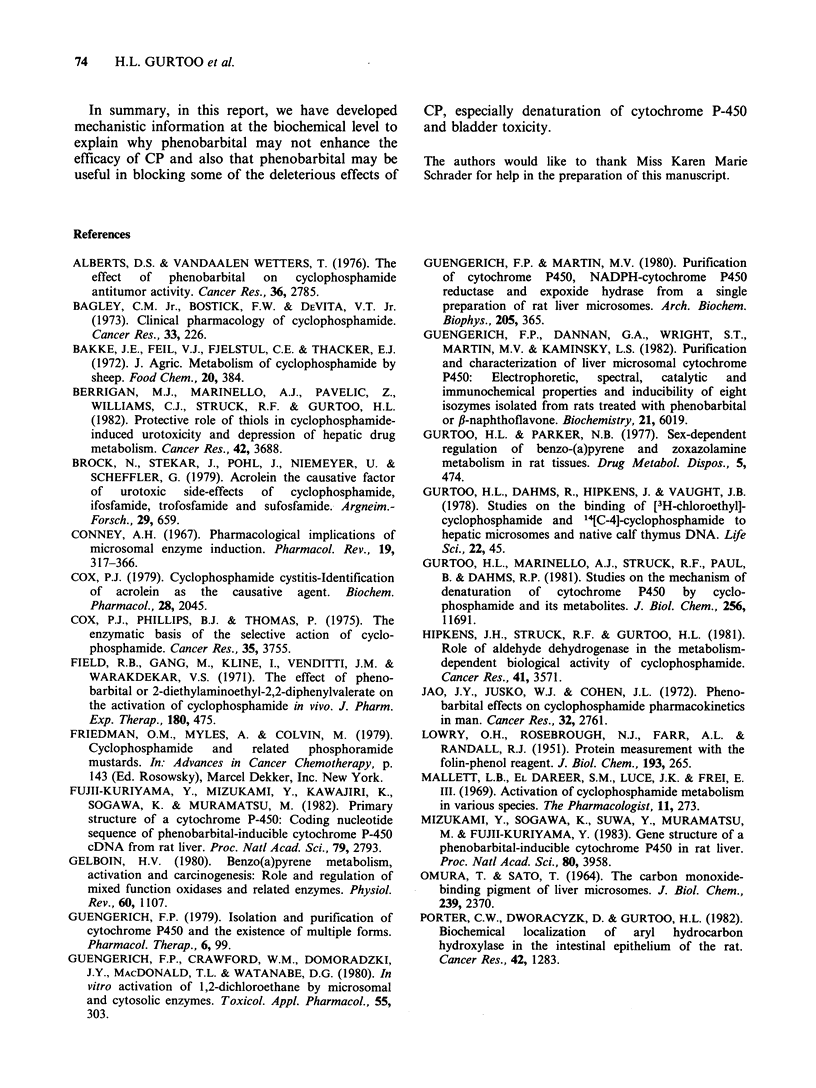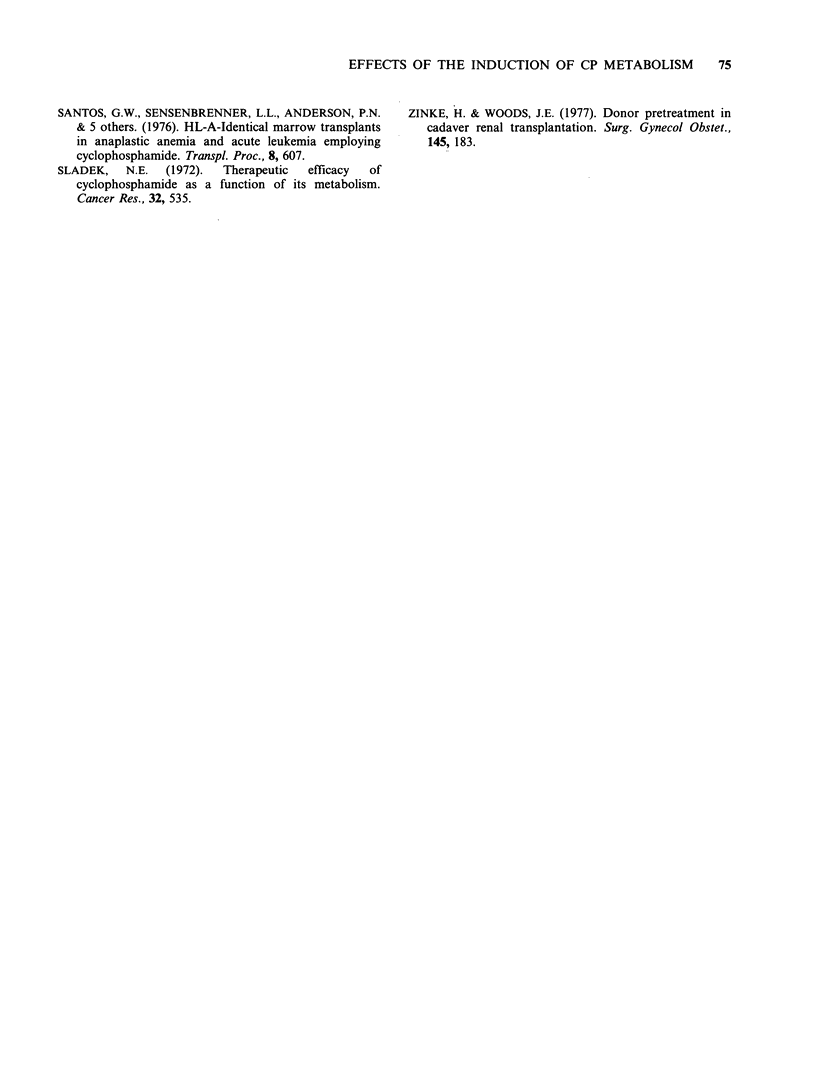# Effects of the induction of hepatic microsomal metabolism on the toxicity of cyclophosphamide.

**DOI:** 10.1038/bjc.1985.10

**Published:** 1985-01

**Authors:** H. L. Gurtoo, S. K. Bansal, Z. Pavelic, R. F. Struck

## Abstract

Cyclophosphamide (CP) administration to rats in a single i.p. dose (200 mg kg-1), while producing urinary bladder toxicity and 30-40% depression of the hepatic microsomal mixed function oxidase (MFO), failed to produce any depression of MFO activities in extrahepatic tissues such as lung, kidney and intestine. Phenobarbital pretreatment of the rats, which is known to enhance hepatic microsomal activation of CP, protected against CP-induced urinary bladder toxicity and the depression of hepatic MFO activities. This protection appears to be, at least in part, related to phenobarbital induction of hepatic cytochrome P-450 isozyme(s) that metabolizes CP to a new metabolite tentatively identified as didechlorodihydroxycyclophosphamide.


*Br. J. Cancer* (1985), **51**, 67–75

### Effects of the induction of hepatic microsomal metabolism on the toxicity of cyclophosphamide

H.L. Gurtoo, S.K. Bansal, Z. Pavelic & R.F. Struck

*Department of Experimental Therapeutics and Grace Cancer Drug Center, Roswell Park Memorial Institute, New York State Department of Health, 666 Elm Street, Buffalo, NY 14263 and Southern Research Institute (RFS), Birmingham, AL 35205, USA.*

**Summary** Cyclophosphamide (CP) administration to rats in a single i.p. dose (200 mg kg$^{-1}$), while producing urinary bladder toxicity and 30–40% depression of the hepatic microsomal mixed function oxidase (MFO), failed to produce any depression of MFO activities in extrahepatic tissues such as lung, kidney and intestine. Phenobarbital pretreatment of the rats, which is known to enhance hepatic microsomal activation of CP, protected against CP-induced urinary bladder toxicity and the depression of hepatic MFO activities. This protection appears to be, at least in part, related to phenobarbital induction of hepatic cytochrome P-450 isozyme(s) that metabolizes CP to a new metabolite tentatively identified as didechlorodihydroxycyclophosphamide.

The microsomal mixed function oxidase system, principally found in the liver, is involved in the activation and detoxification of numerous xenobiotics including carcinogens, drugs and pesticides, and some endogenous bio-chemicals such as steroids and fatty acids. During recent years ample evidence has accumulated to demonstrate the existence in hepatic microsomes of multiple forms of cytochrome P-450, the terminal enzyme of the oxidase system (Guengerich, 1979; Guengerich *et al.*, 1982). This evidence has resulted from the application of gel electrophoresis, immunological, and recombinant DNA techniques in the investigation of cytochrome P-450 multiplicity in hepatic microsomes from several species of animals treated with different types of microsomal enzyme inducers (Guengerich, 1979; Guengerich *et al.*, 1982; Fujii-Kuriyama *et al.*, 1982; Mizukami *et al.*, 1983). Phenobarbital, a commonly used prototype inducer, has been shown to induce at least four different forms of cytochrome P-450 in rats with overlapping but different substrate specificities (Guengerich 1979; Guengerich *et al.*, 1982). Phenobarbital treatment has also been reported to induce cyclophosphamide metabolism in rodents and humans (Jao *et al.*, 1972; Sladek, 1972; Bagley *et al.*, 1973).

Cyclophosphamide (CP), an oxazaphosphorine, is an important drug in the treatment of cancer and certain diseases of immunological aetiology (Friedman *et al.*, 1979). It has also found application in the immunosuppressive preparation of patients for organ and tissue transplantation (Santos *et al.*, 1976; Zinke & Woods, 1977; Friedman *et al.*, 1979).

CP is inactive *per se* and requires microsomal mixed function oxidase-mediated metabolism to activated metabolites capable of binding covalently to nucleic acids and proteins. The commonly accepted scheme of CP metabolism involves intermediate formation of 4-hydroxy-CP which undergoes ring-opening to form aldophosphamide, an isomer of 4-hydroxy-CP (Figure 1). Both 4-hydroxy-CP and aldophosphamide, can be detoxified by cytosolic oxidases/reductases (Cox *et al.*, 1975; Hipkens *et al.*, 1981). Alternatively, aldophosphamide can undergo non-enzymatic $\beta$ elimination to release acrolein and phosphoramide mustard. Acrolein is believed to be responsible for CP-induced haematuria, one of the limiting toxicities of CP, and phosphoramide mustard has been assigned the distinction of being the therapeutically active metabolite of CP and is believed to be responsible for both the antitumour activity and immunosuppression (Friedman *et al.*, 1979; Brock *et al.*, 1979; Cox, 1979; Berrigan *et al.*, 1982).

During the recent years we have reported that, in addition to being responsible for haematuria, acrolein may also denature cytochrome P-450; both effects are believed to result from the reaction of the double bond in acrolein with free sulfhydryl groups in proteins (Gurtoo *et al.*, 1981). While CP was found to depress various mixed function oxidase activities of the liver *in vivo*, effects on cytochrome P-450-mediated activities in extrahepatic tissues were not investigated.

Because phenobarbital induces CP metabolism,

Correspondence: H.L. Gurtoo.
Received 10 May 1984; and in revised form 9 October 1984.

© The Macmillan Press Ltd., 1985

**Figure 1** Hepatic pathways of cyclophosphamide metabolism.
MFO, mixed function oxidase; CP, cyclophosphamide; 4-OHCP, 4-hydroxycyclophosphamide; 4-ketoCP, 4-ketocyclophosphamide; Aldo-P, aldophosphamide; carboxy P, carboxyphosphamide.

one would anticipate some degree of relationship between this induction and the chemotherapeutic and toxic effects of CP. We have investigated this relationship in the rat in the context of the effects of CP on mixed function oxidase in liver and extrahepatic tissue and of the ability of CP to induce haematuria. These investigations have led to suggestions of the participation of novel detoxification pathways of CP metabolism catalyzed by hepatic microsomes from phenobarbital treated rats.

### Materials and methods

Sources of CP and other chemicals needed for enzyme assays have been detailed earlier (Hipkens *et al.*, 1981; Gurtoo *et al.*, 1981; Berrigan *et al.*, 1982).

### *Animals tissue and enzyme preparation*

Male rats (175–225 g) were used in all the studies. Source and housing details are given elsewhere (Berrigan *et al.*, 1982). CP was injected i.p. as a single injection at a dose of 200 mg kg$^{-1}$, and the rats were killed 4 days later. Microsomes or 15,000 *g* supernatant from liver, kidney, lung and intestine were isolated as described previously (Gurtoo & Parker, 1977; Gurtoo *et al.*, 1981; Berrigan *et al.*, 1982; Porter *et al.*, 1982), and the protein content was determined by the method of Lowry *et al.* (1951), using bovine serum albumin as the reference standard. Urinary bladders, when needed, were removed, immersed in 10% buffered formalin and stored for histopathology conducted at a later date.

### *Enzyme assays*

Cytochrome P-450 was determined according to the method of Omura & Sato (1964) using an Aminco DW-2 spectrophotometer. The calculation of cytochrome P-450 content is based on the extinction coefficient of 91 mM$^{-1}$cm$^{-1}$. Aryl hydrocarbon hydroxylase (AHH) and aminopyrine demethylase activities were determined by previously described methods (Gurtoo *et al.*., 1981; Berrigan *et al.*, 1982).

### *Histopathology*

Urinary bladders were fixed in Bouin's solution and sectioned at 5 $\mu$m and the sections for microscopic examination were stained with haematoxylin and eosin (Berrigan *et al.*, 1982).

### *Purification of cytochrome P-450*

Cytochrome P-450 was purified from the hepatic microsomes of phenobarbital pretreated male rats by the method of Guengerich & Martin (1980). Sodium phenobarbital was included in the drinking water as a 0.1% solution for 7 days prior to the sacrifice of the animals. The detergent solubilized hepatic microsomes were subjected to chromatography on octylamino-Sepharose-4B. The cytochrome P-450 band eluted from this column was subjected to DEAE-cellulose chromatography as described by Guengerich & Martin (1980). Although three peaks eluted from this column, only peak $B_2$ was found to metabolize either [chloroethyl-$^3$H]CP or [4-$^{14}$C]CP. The DEAE-cellulose column fractions forming peak $B_2$ were pooled, treated with Bio-beads SM-2 (BioRad Labs., Richmond, CA) to remove the detergent, concentrated and dialyzed against 10 mM Tris-acetate buffer (pH 7.4) containing 1 mM EDTA and 20% glycerol. The purified "Peak $B_2$" cytochrome P-450 was stored in small aliquots at −70°C for up to 6 months without any loss of activity. The specific activity ranged between 15–17 nmol cytochrome P-450 mg$^{-1}$ protein. NADPH-Cytochrome P-450 reductase was also purified from the same batch of solubilized microsomes according to the method of Guengerich & Martin (1980). The specific activity of reductase was 57 units mg$^{-1}$ protein (1 unit = 1 μmol cytochrome C reduced min$^{-1}$).

*Metabolism of CP*

Metabolism of both [chloroethyl-$^3$H]CP and [4-$^{14}$C]CP was studied with hepatic microsomes from untreated, 3-methylcholanthrene treated and phenobarbital treated rats or with reconstituted system containing cytochrome P-450 purified from hepatic microsomes from phenobarbital treated rats. Sodium phenobarbital was administered either i.p. as 2 daily injections (40 mg kg$^{-1}$) for 3 consecutive days or in drinking water as 0.1% solution for 7 days. 3-Methylcholanthrene was administered i.p. as a single daily injection (30 mg kg$^{-1}$) for 3 consecutive days. Control rats received only the vehicle.

*Metabolism by hepatic microsomes*

Hepatic microsomes, one mg protein, isolated from control, 3-methylcholanthrene and phenobarbital treated rats were incubated in the presence or absence of an NADPH-generating system with [chloroethyl-$^3$H]-CP or [4-$^{14}$C]-CP for 15 min as derived before (Berrigan *et al.*, 1982). At the termination of the incubation, the mixture was extracted twice with 3 to 4 volumes of chloroform: isoamyl alcohol (95:5) and an aliquot was counted for the quantitation of the polar metabolite formation.

*Metabolism by the purified Cytochrome P-450*

Both [chloroethyl-$^3$H]CP and [4-$^{14}$C]CP were metabolized in the cytochrome P-450 reconstituted system. The composition of 1 ml of buffered incubation mixture was as follows: 10 μg dilauroyl-phosphatidyl choline (Serdary Research Labs., Ontario, Canada), 0.4 units NADPH-cytochrome P-450 reductase, 0.1 nmole cytochrome P-450, 7.5 μmol $MgCl_2$ and 0.3 μmole [chloroethyl-$^3$H]-CP or [4-$^{14}$C]-CP. The reaction was started with the addition of 0.2 μmol NADPH, and at 20 and 40 min the reaction mixture was fortified with 0.1 nmol cytochrome P-450 and 0.2 μmol NADPH. The reaction was terminated at 60 min by freezing the incubation mixture in dry ice. Depending upon the need, 10 to 15 tubes containing 1 ml of incubation mixture were processed.

For metabolite isolation, as required, incubates were thawed and extracted with chloroform. The aqueous phase was lyophilized and the residue was first extracted with chloroform and then with method. CP metabolites were resolved by silica gel thin layer chromatography in several solvent systems and by HPLC on a partisil PAC column with acetonitrile: methanol gradient. Radioactivity was counted in Aquasol (New England Nuclear, Boston, MA) in a Packard Tri-Carb model 3315 liquid scintillation counter with external standard. Radiochromatographic scanning of silica gel plates was performed with a Packard Model 7220/21 Scanner.

### Results

*Effect of CP on mixed function oxidase activities in extrahepatic tissues*

Investigations were carried out to determine the effects of CP on mixed function oxidase activities in extrahepatic tissues such as kidney, lung and intestine. These tissues were analyzed for the demethylation of aminopyrine and for AHH activity and the results compared with the hepatic activities (Table I). While CP caused a depression of AHH by 37% and aminopyrine demethylase by 30% in the liver, it failed to produce any inhibition of these activities in lungs, kidneys and intestine. Instead, CP was found to increase intestinal AHH activity by about 50%.

*Effects of phenobarbital on CP-induced depression of mixed function oxidase in liver and extrahepatic tissues*

Phenobarbital pretreatment of rats has been reported to induce CP metabolism (Sladek, 1972; Gurtoo *et al.*, 1978). Because of these reports and

**Table I** Comparison of the effects of cyclophosphamide (CP) on mixed function oxidase activity in liver and extrahepatic tissues of the rat[a]

| *MFO activity* | *Treatment* | *Liver mean ± s.e.* | *Tissue* *Lung mean ± s.e.* | *Kidney mean ± s.e.* | *Intestine mean ± s.e.* |
|---|---|---|---|---|---|
| AHH activity[b] | Control | 280±21 (100) | 2.8±0.1 (100) | 12.6±0.4 (100) | 2.6±0.2 (100) |
| | CP | 178±7[d] (63) | 3.1±0.1[e] (110) | 12.1±0.5[e] (96) | 3.9±0.1[d] (150) |
| Aminopyrine Demethylase[c] | Control | 555±35 (100) | 54.0±1.6 (100) | 51.3±1.6 (100) | 64.1±1.8 (100) |
| | CP | 388±10[d] (70) | 58.7±0.2[e] (108) | 51.9±0.5[e] (101) | 65.6±1.2[e] (102) |

[a]Each treatment group contained four rats and each individual result was obtained as a mean of duplicate or triplicate analytical determinations. Similar results were obtained in another experiment.
[b]AHH activity expressed as pmole equivalents of 3-hydroxybenzo(a)pyrene formed mg protein$^{-1}$ min$^{-1}$. The numbers in parenthesis represent percent of control.
[c]Aminopyrine demethylase activity expressed as nmoles formaldehyde formed mg protein$^{-1}$ h$^{-1}$. The numbers in parenthesis represent percent of control.
[d]Significantly different from control values ($P<0.05$).
[e]Statistically insignificant difference from control values ($P=0.05$).

**Table II** Effects of pretreatment with phenobarbital on cyclophosphamide-induced depression of liver mixed function oxidase (MFO) activities[a]

| *Treatment* | *MFO activity*[b] *AHH activity mean ± s.e.* | *Aminopyrine demethylase activity*[c] *mean ± s.e.* |
|---|---|---|
| Control | 280±21 (100) | 555±35 (100) |
| Phenobarbital (PB) | 399±10[d] (142) | 894±22[d] (161) |
| CP alone | 178± 5[d] (63) | 388±11[d] (70) |
| PB+CP | 330± 3[e] (118) | 760±17[d] (137) |

[a]Each treatment group contained four rats and each individual result was obtained as a mean of duplicate or triplicate analytical determinations. Similar results were obtained in another experiment.
[b]AHH activity expressed as pmole equivalents of 3-hydroxybenzo(a)pyrene formed mg protein$^{-1}$ min$^{-1}$. The numbers in parenthesis represent percent of control.
[c]Aminopyrine demethylase activity expressed as nmol formaldehyde formed mg protein$^{-1}$ h$^{-1}$. The numbers in parenthesis represent percent of control.
[d]Significantly different from control values ($P<0.05$).
[e]Statistically insignificant difference from control values ($P=0.05$).

the observations that CP induces metabolism-mediated depression of hepatic cytochrome P-450 and the associated activities, including its own metabolism, it was argued that induction of CP metabolism should lead to an enhancement in CP-induced depression of the mixed function oxidase activities. The result of the studies performed to investigate this relationship are given in Table II.

In accordance with several other reports (Conney, 1967), phenobarbital was found to enhance the microsomal mixed function oxidase activities; it increased aminopyrine demethylase activity by 61% and AHH activity by 42%. In contrast and as expected from previous studies (Gurtoo *et al.*, 1981; Berrigan *et al.*, 1982), CP decreased these mixed function oxidase-mediated activities by 30 and 37%, respectively. However, when CP was combined with phenobarbital pretreatment, phenobarbital failed to produce an enhancement in the depression of these activities; but, on the other hand, the activities of the CP plus phenobarbital group were 37% and 20%, respectively, higher than the control group. Even if the activities of the CP plus phenobarbital group are compared with phenobarbital treatment alone, the presence of phenobarbital decreased the activities half as much as CP treatment alone decreased these activities in the control animals (aminopyrine demethylase, 15% *vs* 30%; AHH, 17% *vs* 37%). These results suggest that phenobarbital probably induces various forms of cytochrome P-450 isozymes that participate in the activation as well as in the detoxification of CP.

Phenobarbital pretreatment also failed to render CP depressant to the mixed function oxidase activities in extrahepatic tissues such as kidney, lungs and intestine.

### *Effect of phenobarbital on the urotoxicity of CP*

As reported previously (Berrigan *et al.*, 1982) CP treatment produced acute histopathologic changes in the urinary bladders of rats examined 4 days after CP administration. These changes included thickening of the bladder mucosa, ulceration, and haemorrhagic and necrotic lesions in the mucosa. Bloody exudate containing cellular debris, fibrin and inflammatory cells was seen in the lumen. In addition, focal changes of the seemingly intact mucosa, including thinning, atypia and Karyorrhexis were also observed. Diffuse oedematous and haemorrhagic changes were also seen in the submucosa.

Pretreatment of the rats with phenobarbital alone or treatment of the rats with saline (control) had no demonstrable effect on the urinary bladder histology. However, when CP was administered to rats pretreated with phenobarbital, only one of four rats showed slight atypia and vacuolization of epithelial cells of the mucosa and slight oedematous and haemorrhagic changes in the submucosa. These changes were mild relative to CP treatment alone. These results clearly demonstrate the ability of phenobarbital to afford significant protection against CP-induced bladder damage.

*Metabolism of CP by hepatic microsomes*

The effects of phenobarbital and 3-methylcholanthrene pretreatment on the formation of the polar metabolites of CP by the hepatic microsomes are shown in Table III. Simultaneous presence of both NADPH and hepatic microsomes was essential for the formation of the polar metabolites. More [$^{3}$H]-labelled metabolites than [$^{14}$C]-labelled metabolites were recovered in the aqueous phase following chloroform extraction of the incubation mixture. While 3-methylcholanthrene pretreatment of the rats resulted in a mild depression of the metabolism, phenobarbital pretreatment enhanced the polar metabolite formation 10 to 14-fold. Thin layer chromatographic and HPLC analyses of the polar fractions derived from larger incubations repeatedly revealed the presence of a polar metabolite, which has been tentatively identified as didechloro-dihydroxy-CP (described below). This was not formed in incubations lacking in NADPH and was produced in only negligible amounts by hepatic microsomes from control rats, whereas hepatic microsomes from phenobarbital treated rats produced large quantities of this metabolite.

The data on phenobarbital (Table III) also suggest that of the total [$^{3}$H]-labelled polar metabolites ~40% contain [$^{14}$C]-label as well. This is compatible with the formation of didechlorodihydroxy-CP as at least one of the major polar metabolites of CP. The only other CP metabolite with only [$^{14}$C]-label would be acrolein (see Figure 1) which is expected to be removed into the chloroform phase during the extraction of the incubation mixture. Using [$^{14}$C]-acrolein we found that under the experimental conditions ~90% of acrolein is extracted by the chloroform:isoamyl alcohol (95:5) mixture used to extract the incubation mixtures. The difference between [chloroethyl-$^{3}$H]-CP and [4-$^{14}$C]-CP radioactivity in the aqueous phase is attributable to the presence of phosphoramide mustard and its degradation products (see Figure 1).

*Metabolism of CP by the purified cytochrome P-450 from phenobarbital-treated rats*

Cytochrome P-450, purified from phenobarbital treated rats, was employed in the reconstituted system to investigate the metabolism of both [chloroethyl-$^{3}$H]-CP and [4-$^{14}$C]-CP. As described earlier, the incubations were extracted with chloroform, the aqueous phase was lyophilized, and the residue extracted sequentially with chloroform and methanol.

Radioactivity measurements demonstrated that chloroform extracts consistently contained 20–35%

**Table III** Metabolism of [$^{3}$H-chlorethyl]cyclophosphamide ($^{3}$H-CP) and [4-$^{14}$C]-cyclophosphamide ($^{14}$C-CP) by hepatic microsomes from control and induced rats

| | *Formation of polar metabolites of CP*[a] *nmol metabolites mg$^{-1}$ microsomal protein 15 min$^{-1}$* | |
|---|---|---|
| *Microsomes* | *$^{14}$C-CP* | *$^{3}$H-CP* |
| Control | 2.93±0.01 | 5.21±0.07 |
| 3MC-induced[b] | 1.96±0.03 | 2.84±0.05 |
| PB-induced[b] | 30.34±0.09 | 71.58±1.48 |

[a]Mean+s.e. of four determinations. Values obtained in the absence of NADPH have been subtracted and were 1.46±0.05 for $^{14}$C-CP and 3.76 ±0.09 for $^{3}$H-CP; no significant differences between control, 3MC-induced and PB-induced microsomes were observed in the absence of NADPH.

[b]3MC, 3-methylcholanthrene; PB, phenobarbital.

of the total radioactivity while methanol extract contained 60–70% of the total radioactivity in the incubates. The remaining radioactivity (5–10%) was associated with the methanol-extracted residue. Using authentic standards, ~90% of the chloroform-extractable radioactivity cochromatographed on silica gel thin layer plates, developed with acetone:chloroform (3:1), with unmetabolized CP, while small amounts cochromatographed with alcophosphamide (~5%), aldophosphamide cyanohydrin (~4%) and dechloroethyl CP (~2%).

Methanol extracts, after treatment with diazomethane to stabilize phosphoramide mustard as its methyl ester, revealed on thin layer chromatography (chloroform:methanol, 9:1) only trace amounts of phosphoramide mustard, while the major amount of radiactivity remained at the origin of the silica gel plates. The high polarity of the major radioactivite band was established by TLC in different solvent systems.

Side-by-side TLC of methanol extracts of incubates from [chloroethyl-$^3$H]-CP and [4-$^{14}$C]-CP in a variety of TLC solvent systems (chloroform: methanol [1:1] and [1:2], methanol alone, and methanol:water [9:10] demonstrated by radioscanning that the $R_f$s of the major radioactive component in extracts of both labels were identical. Diazomethane treatment of both the [$^3$H] and [$^{14}$C]-labelled major metabolite, either in total methanol extracts or after TLC isolation, had no effect on TLC mobility; this property demonstrates the absence of an acidic function in the metabolite and indicates that the metabolite has retained the oxazaphosphorine ring intact.

Consideration of the foregoing data led to the tentative identification of the metabolite as didechlorodihydroxy-CP. Such a structure would be expected to be polar and to be unaffected by diazomethane and would retain both [$^3$H] and [$^{14}$C] labels.

Likely routes to didechlorodihydroxy-CP would involve selective hydrolytic displacement of the chlorine groups in CP without concomitant hydrolysis of phosphoramide or P(V) alkyl ester bonds. Since phosphoramides are sensitive to acid hydrolysis but resist base hydrolysis, synthesis was attempted by selective chlorine displacement from [chloroethyl-$^3$H]-CP by hydroxide ion in dilute potassium hydroxide. The method was successful, and the tritiated dihydroxy analog of CP was isolated by TLC. Structural confirmation was obtained by mass spectral analysis using the fast atom bombardment technique. In the normal (positive) mode, an intense peak of $m/z$ 225 (strongest peak in spectrum) was observed ($[M+1]^+$). In the negative mode, a peak of $m/z$ 223 (strongest peak in spectrum) was observed ($[M-1]^-$).

Synthetic 2-[bis(2-hydroxyethyl)amino]tetrahydro-2H-1,3,2-oxazaphosphorine 2-oxide (didechlorodihydroxy-CP) was compared by TLC with the major metabolite generated in incubates of the isolated cytochrome P-450 fraction with [chloroethyl-$^3$H]-CP and [4-$^{14}$C]-CP. Identical $R_f$s were obtained in different solvent systems upon side-by-side TLC on silica gel (Table IV).

**Table IV** $R_f$ values of [$^3$H]-labelled synthetic didechlorodihydroxy CP and of the major methanol extractable metabolite of [chloroethyl-$^3$H]CP and [4-$^{14}$C]CP.

| *Solvent* | $R_f$ |
|---|---|
| Acetone | 0.0 |
| Methanol | 0.35 |
| Mehanol (2-developments) | 0.60 |
| Ethanol | 0.08 |
| Ethanol (2-developments) | 0.15 |
| Methanol:water (9:1) | 0.50 |
| Methanol:water (1:1) | 0.60 |

Additional confirmation was sought by HPLC. HPLC of the synthetic [$^3$H]-didechlorodihydroxy-CP and the TLC-isolated major metabolite from incubates of purified cytochrome P-450 with [chloroethyl-$^3$H]-CP and [4-$^{14}$C]-CP, under precisely identical conditions on the same day with collection of 40 1-ml fractions followed by radioassay, gave the major radioactivity in the same fraction (No. 19) in every case. In order to eliminate the possibility of slight variation in HPLC retention times of the major radioactive metabolite in [$^3$H]- and [$^{14}$C]-incubates, [$^3$H]- and [$^{14}$C]-TLC-isolated fractions were mixed and separated by HPLC with fraction collection and radioassay for [$^3$H] and [$^{14}$C]. The major radioactivity observed in both [$^3$H] and [$^{14}$C] channels appeared in the same fraction (No. 18).

Consideration of the collected data on the common, major [$^3$H]- and [$^{14}$C]-labelled metabolite (high polarity; unaffected by diazomethane; common to both labels; identical by side-by-side TLC in several solvent systems and by precisely-identical HPLC profile to synthetic didechlorodihydroxy-CP; identical by co-elution HPLC) strongly suggests that the metabolite is didechlorodihydroxy-CP. Further confirmation was obtained by converting the TLC-purified metabolite to CP by the chlorinating agent thionyl chloride.

### Discussion

Recently, we reported that the metabolism of CP is accompanied by inactivation of hepatic microsomal cytochrome P-450 (Gurtoo *et al.*, 1981). Our studies have incriminated cyclophosphamide metabolite acrolein in the depression of liver cytochrome P-450 and, in agreement with other reports (Brock *et al.*, 1979; Cox, 1979), have also implicated acrolein in the causation of CP-associated haematuria and bladder toxicity (Berrigan *et al.*, 1982). However, the present investigations have demonstrated that while treatment of rats with CP depresses liver mixed function oxidase activities, no such depression of similar activities is produced in the extrahepatic tissues. This result is compatible with the likely possibility that most of the CP is metabolized during its first pass through the liver and sufficient amounts of CP, capable of producing deleterious amounts of acrolein, do not reach extrahepatic tissues. This interpretation would mean that cytochrome P-450 in extrahepatic tissues is probably equally sensitive to the deleterious effects of the CP metabolite acrolein.

Phenobarbital is a well known inducer of various forms of cytochrome P-450 (Guengerich *et al.*, 1982) and associated activities, including the metabolism of CP (Sladek, 1972; Gurtoo *et al.*, 1978). Sladek (1972) reported that phenobarbital pretreatment enhanced the hepatic microsomal metabolism of CP to its alkylating metabolites; it decreased the Km and enhanced the Vmax by about 7-fold. However, various attempts to enhance the chemotherapeutic activity and whole body toxicity of CP have produced conflicting results. Sladek (1972) reported that phenobarbital pretreatment of male rats did not alter the therapeutic activity of CP against Walker 256 carcinosarcoma. In other studies involving mice, Field *et al.* (1971) found that while phenobarbital pretreatment accelerated the production of alkylating metabolites of CP, duration of antileukaemic activity against L1210 leukaemic cells was shortened by phenobarbital. Alberts & Wetters (1976) found a constant 90% (1-log) reduction in the toxicity of CP to P388 leukaemic colony forming units in mice. In various studies on the pharmacokinetics of CP in patients, phenobarbital was found to accelarate the metabolism of CP and reduce its half-life (Mallett *et al.*, 1969; Jao *et al.*, 1972; Bagley *et al.*, 1973). One report even suggested that this increased activation in humans may offer therapeutic advantage in the treatment of cancer (Mallett *et al.*, 1969). However, Jao *et al.* (1972) found that phenobarbital, while increasing the rate of biotransformation of CP 2 to 3-fold, had no quantitatively significant effect on the distribution and renal excretion of CP. These investigators concluded that phenobarbital should marginally enhance the efficacy and toxicity of CP in man.

In the present report, we have demonstrated that phenobarbital, which was earlier found to enhance the formation of protein- and DNA-binding metabolites of CP formed *in vitro* by the hepatic microsomes (Gurtoo *et al.*, 1978), failed to enhance CP-induced depression of the hepatic mixed function oxidase and also protected against the toxicity of CP to the urinary bladder. This enigmatous observation led to the rationalization that phenobarbital, which is known to induce various forms of cytochrome P-450, was inducing both the activation and the detoxification of CP. If this was true, it would explain the reported effects of phenobarbital pretreatment on the therapeutic efficacy and toxicity of CP: increase in the formation of alkylating metabolites of CP both *in vivo* and *in vitro*; decrease in $t_{\frac{1}{2}}$ of CP, and minimal effect on the toxicity and therapeutic activity of CP.

In fulfilment of the requirement to demonstrate enhancement in the detoxification of CP by phenobarbital, we demonstrated the formation of a very polar metabolite, tentatively identified as didechlorodihydroxy-CP. This metabolite was found to be one of the major metabolites of CP catalyzed by cytochrome P-450 isolated from phenobarbital treated rats. This biotransformation would essentially yield a detoxified product of CP. We do not know at this stage whether the monodechlorinated metabolite exists or whether 4-hydroxy CP could be further metabolized to form didechlorinated products. Further metabolism of the primary metabolites by the mixed function oxidase system is a well established metabolic route in the biotransformation of several polycyclic aromatic hydrocarbons (Gelboin, 1980).

A precedent for replacement of chlorine by the hydroxyl group in CP by cytochrome P-450 can be found in studies on mixed function oxidation of 1,2-dichloroethane to 2-chloroethanol by Guengerich *et al.* (1980). Additionally, in studies on the urinary metabolites of CP in sheep, Bakke *et al.* (1972) identified a minor metabolite whose structure is similar to that of the cytochrome P-450 metabolite. Mass spectral analysis led to the suggestion that the sheep metabolite was 5- or 6-keto-didechlorodihydroxy-CP. The sheep metabolite establishes a precedent for replacement of chlorine by the hydroxyl group in CP *in vivo*.

The existence of a phenobarbital-inducible cytochrome P-450 isozyme that is capable of dechlorinating CP is consistent with the inability of phenobarbital to appreciably enhance the experimental antitumour effects of CP.

In summary, in this report, we have developed mechanistic information at the biochemical level to explain why phenobarbital may not enhance the efficacy of CP and also that phenobarbital may be useful in blocking some of the deleterious effects of CP, especially denaturation of cytochrome P-450 and bladder toxicity.

The authors would like to thank Miss Karen Marie Schrader for help in the preparation of this manuscript.

### References

ALBERTS, D.S. & VANDAALEN WETTERS, T. (1976). The effect of phenobarbital on cyclophosphamide antitumor activity. *Cancer Res.*, **36**, 2785.

BAGLEY, C.M. Jr., BOSTICK, F.W. & DEVITA, V.T. Jr. (1973). Clinical pharmacology of cyclophosphamide. *Cancer Res.*, **33**, 226.

BAKKE, J.E., FEIL, V.J., FJELSTUL, C.E. & THACKER, E.J. (1972). J. Agric. Metabolism of cyclophosphamide by sheep. *Food Chem.*, **20**, 384.

BERRIGAN, M.J., MARINELLO, A.J., PAVELIC, Z., WILLIAMS, C.J., STRUCK, R.F. & GURTOO, H.L. (1982). Protective role of thiols in cyclophosphamide-induced urotoxicity and depression of hepatic drug metabolism. *Cancer Res.*, **42**, 3688.

BROCK, N., STEKAR, J., POHL, J., NIEMEYER, U. & SCHEFFLER, G. (1979). Acrolein the causative factor of urotoxic side-effects of cyclophosphamide, ifosfamide, trofosfamide and sufosfamide. *Argneim.-Forsch.*, **29**, 659.

CONNEY, A.H. (1967). Pharmacological implications of microsomal enzyme induction. *Pharmacol. Rev.*, **19**, 317–366.

COX, P.J. (1979). Cyclophosphamide cystitis-Identification of acrolein as the causative agent. *Biochem. Pharmacol.*, **28**, 2045.

COX, P.J., PHILLIPS, B.J. & THOMAS, P. (1975). The enzymatic basis of the selective action of cyclophosphamide. *Cancer Res.*, **35**, 3755.

FIELD, R.B., GANG, M., KLINE, I., VENDITTI, J.M. & WARAKDEKAR, V.S. (1971). The effect of phenobarbital or 2-diethylaminoethyl-2,2-diphenylvalerate on the activation of cyclophosphamide *in vivo*. *J. Pharm. Exp. Therap.*, **180**, 475.

FRIEDMAN, O.M., MYLES, A. & COLVIN, M. (1979). Cyclophosphamide and related phosphoramide mustards. *In: Advances in Cancer Chemotherapy*, p. 143 (Ed. Rosowsky), Marcel Dekker, Inc. New York.

FUJII-KURIYAMA, Y., MIZUKAMI, Y., KAWAJIRI, K., SOGAWA, K. & MURAMATSU, M. (1982). Primary structure of a cytochrome P-450: Coding nucleotide sequence of phenobarbital-inducible cytochrome P-450 cDNA from rat liver. *Proc. Natl Acad. Sci.*, **79**, 2793.

GELBOIN, H.V. (1980). Benzo(a)pyrene metabolism, activation and carcinogenesis: Role and regulation of mixed function oxidases and related enzymes. *Physiol. Rev.*, **60**, 1107.

GUENGERICH, F.P. (1979). Isolation and purification of cytochrome P450 and the existence of multiple forms. *Pharmacol. Therap.*, **6**, 99.

GUENGERICH, F.P., CRAWFORD, W.M., DOMORADZKI, J.Y., MACDONALD, T.L. & WATANABE, D.G. (1980). *In vitro* activation of 1,2-dichloroethane by microsomal and cytosolic enzymes. *Toxicol. Appl. Pharmacol.*, **55**, 303.

GUENGERICH, F.P. & MARTIN, M.V. (1980). Purification of cytochrome P450, NADPH-cytochrome P450 reductase and expoxide hydrase from a single preparation of rat liver microsomes. *Arch. Biochem. Biophys.*, **205**, 365.

GUENGERICH, F.P., DANNAN, G.A., WRIGHT, S.T., MARTIN, M.V. & KAMINSKY, L.S. (1982). Purification and characterization of liver microsomal cytochrome P450: Electrophoretic, spectral, catalytic and immunochemical properties and inducibility of eight isozymes isolated from rats treated with phenobarbital or $\beta$-naphthoflavone. *Biochemistry*, **21**, 6019.

GURTOO, H.L. & PARKER, N.B. (1977). Sex-dependent regulation of benzo-(a)pyrene and zoxazolamine metabolism in rat tissues. *Drug Metabol. Dispos.*, **5**, 474.

GURTOO, H.L., DAHMS, R., HIPKENS, J. & VAUGHT, J.B. (1978). Studies on the binding of [$^3$H-chloroethyl]-cyclophosphamide and $^{14}$[C-4]-cyclophosphamide to hepatic microsomes and native calf thymus DNA. *Life Sci.*, **22**, 45.

GURTOO, H.L., MARINELLO, A.J., STRUCK, R.F., PAUL, B. & DAHMS, R.P. (1981). Studies on the mechanism of denaturation of cytochrome P450 by cyclophosphamide and its metabolites. *J. Biol. Chem.*, **256**, 11691.

HIPKENS, J.H., STRUCK, R.F. & GURTOO, H.L. (1981). Role of aldehyde dehydrogenase in the metabolism-dependent biological activity of cyclophosphamide. *Cancer Res.*, **41**, 3571.

JAO, J.Y., JUSKO, W.J. & COHEN, J.L. (1972). Phenobarbital effects on cyclophosphamide pharmacokinetics in man. *Cancer Res.*, **32**, 2761.

LOWRY, O.H., ROSEBROUGH, N.J., FARR, A.L. & RANDALL, R.J. (1951). Protein measurement with the folin-phenol reagent. *J. Biol. Chem.*, **193**, 265.

MALLETT, L.B., EL DAREER, S.M., LUCE, J.K. & FREI, E. III. (1969). Activation of cyclophosphamide metabolism in various species. *The Pharmacologist*, **11**, 273.

MIZUKAMI, Y., SOGAWA, K., SUWA, Y., MURAMATSU, M. & FUJII-KURIYAMA, Y. (1983). Gene structure of a phenobarbital-inducible cytochrome P450 in rat liver. *Proc. Natl Acad. Sci.*, **80**, 3958.

OMURA, T. & SATO, T. (1964). The carbon monoxide-binding pigment of liver microsomes. *J. Biol. Chem.*, **239**, 2370.

PORTER, C.W., DWORACYZK, D. & GURTOO, H.L. (1982). Biochemical localization of aryl hydrocarbon hydroxylase in the intestinal epithelium of the rat. *Cancer Res.*, **42**, 1283.

SANTOS, G.W., SENSENBRENNER, L.L., ANDERSON, P.N. & 5 others. (1976). HL-A-Identical marrow transplants in anaplastic anemia and acute leukemia employing cyclophosphamide. *Transpl. Proc.*, **8**, 607.

SLADEK, N.E. (1972). Therapeutic efficacy of cyclophosphamide as a function of its metabolism. *Cancer Res.*, **32**, 535.

ZINKE, H. & WOODS, J.E. (1977). Donor pretreatment in cadaver renal transplantation. *Surg. Gynecol Obstet.*, **145**, 183.